# Supplementary material for: Characterization of a Lytic Bacteriophage and Demonstration of Its Combined Lytic Effect with a K2 Depolymerase on the Hypervirulent Klebsiella pneumoniae Strain 52145
Source: Microorganisms. 2023 Mar 6;11(3):669. doi: 10.3390/microorganisms11030669 (PMC10051899; doi:10.3390/microorganisms11030669)
Supplement: Supplementary file 1 [file microorganisms-11-00669-s001.zip › Supplementary Table S1.pdf]

Klebsiella phage vB_KpnD_PeteCarol	98%	95.94%	OL539448.1
Klebsiella phage GML-KpCol1	98%	96.29%	NC_047907.1
Klebsiella phage PhiKpNIH-10	99%	95.12%	MN395285.1
Klebsiella phage MezzoGao	98%	95.70%	NC_047850.1
Klebsiella phage cp39	97%	95.71%	OX335432.1
Klebsiella phage cp45	97%	95.71%	OX335380.1
Klebsiella phage mtp24	91%	96.41%	OX335371.1
Klebsiella phage mtp23	91%	96.35%	OX335421.1
Klebsiella phage vB_KpnS-VAC7	93%	96.05%	MZ428225.1
Klebsiella phage pK8	99%	95.38%	OL702938.1
Klebsiella phage vB_KpnS_15-38_KLPPOU149	97%	95.52%	NC_049842.1
Bacteriophage sp.	97%	95.27%	OP075789.1
Klebsiella phage PWKp14	97%	96.91%	MZ634345.1
Klebsiella phage B1	92%	96.15%	MW672037.1
Klebsiella phage vB_KpnS-VAC4	92%	95.49%	MZ428222.1
Klebsiella phage mtp19	96%	97.01%	OX335422.1
Klebsiella phage Sushi	94%	96.02%	NC_028774.1
Klebsiella phage Sanco	93%	96.02%	MK618657.1
Klebsiella phage mtp28	97%	97.17%	OX335385.1
Klebsiella phage mtp27	97%	97.17%	OX335384.1
Klebsiella phage cp41	97%	96.59%	OX335412.1
Klebsiella phage mtp20	91%	95.24%	OX335435.1
Klebsiella phage mtp21	91%	95.24%	OX335429.1
Klebsiella phage vB_KshKPC-M	88%	94.13%	ON489264.2
Klebsiella phage NJR15	95%	95.72%	NC_048044.1
Klebsiella phage mtp29	97%	95.31%	OX335388.1
Klebsiella phage Skenny	95%	94.12%	NC_049841.1
Klebsiella phage KPN N141	97%	96.12%	NC_047841.1
Klebsiella phage vB_KpnS-VAC111	96%	95.73%	ON881905.1
Klebsiella pneumoniae phage	96%	96.30%	OU342755.1
Klebsiella phage vB_KpnS_SegesCirculi	88%	91.54%	NC_049833.1
Klebsiella phage KpKT21phi1	96%	95.86%	NC_048143.1

Klebsiella phage PhiKpNIH-2	89%	95.45%	NC_049845.1
Klebsiella phage NJS1	92%	96.87%	NC_048024.1
Klebsiella phage phiW14	92%	96.62%	OK655936.1
Klebsiella phage vB_KpnS_2811	97%	96.17%	LR757892.1
Klebsiella phage mtp3	94%	96.47%	OX335404.1
Klebsiella phage mtp9	94%	96.47%	OX335400.1
Klebsiella phage mtp2	94%	96.47%	OX335370.1
Klebsiella phage mtp10	94%	96.47%	OX335369.1
Klebsiella phage NJS2	94%	96.18%	NC_048043.1
Klebsiella phage GZ9	88%	95.70%	OP267563.1
Klebsiella phage mtp13	93%	95.54%	OX335430.1
Klebsiella phage PSKP16	86%	95.42%	OW251746.1
Klebsiella phage vB_KpnS-VAC70	86%	95.22%	MZ571831.1
Klebsiella phage ABTNL-2	94%	95.20%	MZ221764.1
Klebsiella phage mtp30	96%	96.80%	OX335411.1
Klebsiella phage JY917	70%	96.46%	NC_049843.1
Klebsiella phage vB_KpnS-VAC110	83%	96.46%	OM032871.1
Klebsiella phage vB_KpnS-VAC113	86%	96.46%	MZ571834.1
Klebsiella phage cp1	91%	94.95%	OX335417.1
Klebsiella phage cp2	91%	94.95%	OX335393.1
Klebsiella phage BUCT556A	89%	96.11%	MZ172460.1
Klebsiella phage NPat	90%	96.29%	OM938991.1
Klebsiella phage mtp12	92%	94.59%	OX335386.1
Klebsiella phage vB_Kp_IME328	91%	96.08%	OK138555.1
Klebsiella phage KP36	88%	95.82%	JF501022.1
Klebsiella phage KpS4	88%	95.83%	OL674249.1
Klebsiella phage KP1801	89%	95.79%	NC_049848.1
Klebsiella phage vB_KpnS-VAC112	88%	95.78%	MZ571833.1
Klebsiella phage mtp25	98%	95.53%	OX335391.1
Klebsiella phage BMac	90%	96.61%	OM938992.1
Klebsiella phage 1513	89%	95.52%	KP658157.1
Klebsiella phage RAD2	91%	95.30%	NC_055956.1
Klebsiella phage Sin4	91%	93.52%	NC_049847.1
Klebsiella phage vB_KpnD_FairDinkum	94%	96.31%	OL539460.1

Klebsiella phage GH-K3	72699	88%	96.08%	NC_048162.1
Klebsiella phage vB_KpnS-VAC8	74011	93%	96.23%	MZ428226.1
Klebsiella phage KL	72146	92%	92.91%	NC_049838.1
Klebsiella phage VLCpiD7c	68142	89%	94.13%	ON602735.1
Klebsiella phage vB_kpnS-VAC10	75376	92%	95.25%	MZ428227.1
Klebsiella phage 13	60927	76%	96.01%	NC_049844.1
Klebsiella phage TSK1	70554	86%	96.06%	NC_048126.1
Klebsiella phage VLCpiD7a	68315	86%	96.66%	ON602741.1
Klebsiella phage vB_KvaS_F1M1D	71844	89%	95.86%	OL744210.1
Caudovirales sp.	43814	59%	92.57%	BK048394.1
Klebsiella phage NJS3	75015	92%	96.25%	MH633486.1
Klebsiella phage IME268	70086	87%	96.32%	MZ398242.1
Klebsiella phage MMBB	65189	87%	95.08%	MT894005.1
Klebsiella phage vB_KpnS_FZ10	76402	94%	96.18%	NC_049840.1
Klebsiella phage TAH8	76640	94%	96.53%	NC_048042.1
Klebsiella phage Sweeny	75189	96%	96.52%	NC_049839.1
Klebsiella phage vB_KvaS_F2M1D	69399	88%	94.74%	OL744213.1
Klebsiella phage vB_KpnS-VAC11	73479	93%	94.74%	MZ428228.1
Klebsiella phage PWKp15	76123	95%	95.46%	MZ634346.1
Klebsiella phage mtp18	69967	90%	94.56%	OX335423.1
Klebsiella phage mtp17	69972	90%	94.55%	OX335439.1
Klebsiella phage PKP126	48085	84%	92.31%	KR269719.1
Klebsiella phage KLPN1	60996	85%	94.10%	KR262148.1
Klebsiella phage mtp31	76894	95%	92.75%	OX335387.1
Caudovirales sp.	19326	24%	95.13%	BK020750.1
Klebsiella phage cp3	72201	90%	96.37%	OX335383.1
Klebsiella phage cp5	72419	90%	96.37%	OX335381.1
Klebsiella phage P528	43646	89%	84.27%	MW021764.1
Klebsiella phage vB_KpnS-MUC-5.2	70933	89%	95.37%	OM687893.1
Bacteriophage sp.	46646	82%	91.37%	OP072329.1
Klebsiella phage VLCpiD7b	70993	88%	96.10%	ON602750.1
Klebsiella phage vB_KpnS-VAC5	73069	93%	94.38%	MZ428223.1
Klebsiella phage vB_Kpn-VAC111	25415	32%	95.75%	MZ612110.1
Klebsiella phage Shelby	70348	88%	96.94%	NC_049846.1
