# Supplementary material for: Characterization of a Lytic Bacteriophage and Demonstration of Its Combined Lytic Effect with a K2 Depolymerase on the Hypervirulent Klebsiella pneumoniae Strain 52145
Source: Microorganisms. 2023 Mar 6;11(3):669. doi: 10.3390/microorganisms11030669 (PMC10051899; doi:10.3390/microorganisms11030669)
Supplement: Supplementary file 1 [file microorganisms-11-00669-s001.zip › Supplementary Table S2.pdf]

tail fiber protein [Klebsiella phage GML-KpCol1]	100%	95.37%	972	YP_009796909.1
tail fiber protein [Klebsiella phage vB_KpnS-VAC7]	100%	92.60%	973	QZE50811.1
putative tail fiber [Klebsiella phage vB_KpnS_15-38_KLPPOU149]	100%	92.49%	971	YP_009903158.1
surface protein [Klebsiella phage pK8]	100%	91.26%	973	UJQ43530.1
hypothetical protein HOS01_gp19 [Klebsiella phage MezzoGao]	100%	89.62%	973	YP_009792117.1
tail fiber protein [Bacteriophage sp.]	98%	89.95%	965	UWI24671.1
putative tail fiber protein [Klebsiella phage vB_KpnD_PeteCarol]	100%	88.28%	973	UGO52438.1
hypothetical protein [Klebsiella phage PhiKpNIH-10]	100%	88.80%	973	QHB49608.1
tail fiber protein [Klebsiella phage vB_Kpn-VAC111]	98%	87.16%	980	UEP19876.1
putative tail fiber protein [Klebsiella phage NJS2]	100%	87.86%	971	YP_009808730.1
putative tail fiber protein [Klebsiella phage NJS1]	100%	84.49%	992	YP_009807418.1
tail fiber protein [Klebsiella phage Sweeny]	100%	82.34%	917	YP_009902937.1
tail fiber protein [Klebsiella phage vB_KpnS-VAC4]	100%	79.14%	875	QZE50497.1
tail fiber protein [Klebsiella phage KL]	91%	88.29%	893	YP_009902881.1
TPA: tail fiber domain-containing protein [Klebsiella pneumoniae]	92%	87.00%	893	HBS5944437.1
putative tail fiber protein [Klebsiella phage PWKp15]	92%	87.53%	892	UJD05288.1
tail fiber protein [Klebsiella phage ABTNL-2]	92%	87.36%	875	QWT56414.1
putative tail fiber protein [Klebsiella phage vB_KpnD_FairDinkum]	92%	87.05%	875	UGO53464.1
tail fiber protein [Klebsiella phage KPN N141]	91%	85.01%	879	YP_009791648.1
tail fiber domain-containing protein [Klebsiella pneumoniae]	91%	85.54%	883	WP_232804533.1
tail fiber protein [Klebsiella phage vB_KpnS-VAC5]	91%	86.54%	865	QZE50562.1
tail fiber protein [Klebsiella phage Solomon]	100%	65.62%	893	QPB09482.1
tail fiber protein [Klebsiella phage vB_KpnS-VAC6]	92%	79.89%	892	QZE50655.1

tail fiber protein [Klebsiella phage vB_KpnS_2811]	92%	80.35%	894	CAA8012772.1
tail fiber domain-containing protein [Klebsiella phage vB_KpnS_Domnhall]	91%	79.76%	882	YP_009902582.1
putative tail fiber protein [Klebsiella phage vB_KpnS_KingDDD]	92%	79.62%	892	QEG12391.1
putative tail fiber protein [Klebsiella phage vB_KpnS_Alina]	92%	79.49%	892	YP_009902756.1
tail fiber domain-containing protein [Klebsiella phage Skenny]	91%	79.14%	892	YP_009903057.1
tail fiber domain-containing protein [Klebsiella phage vB_KpnS_Call]	91%	78.69%	882	YP_009902674.1
putative tail fiber protein [Klebsiella phage PWKp20]	92%	78.82%	892	UJD06094.1
tail fiber domain-containing protein [Klebsiella phage vB_KpnS_IMGroot]	92%	78.69%	892	YP_009902495.1
putative tail fiber protein [Klebsiella phage P528]	92%	78.82%	892	QPX75198.1
putative tail fiber protein [Klebsiella phage vB_KpnS_Penguinator]	92%	78.69%	892	QEG13412.1
putative tail fiber protein [Klebsiella phage vB_KpnS_SegesCirculi]	92%	78.15%	892	YP_009902416.1
tail fiber protein [Klebsiella phage vB_KpnS-VAC11]	92%	77.30%	884	QZE50981.1
tail fiber protein [Klebsiella phage PWKp14]	70%	78.70%	690	UJD05202.1
putative tail fiber protein [Klebsiella phage NJR15]	92%	74.20%	893	YP_009808806.1
tail fiber protein [Klebsiella phage vB_kpnS-VAC2]	84%	73.77%	892	QZE50403.1
putative tail fiber protein [Klebsiella phage NJS3]	93%	71.62%	893	AXQ68100.1
putative tail fiber protein [Klebsiella phage TAH8]	92%	71.12%	894	YP_009808652.1
tail fiber protein [Klebsiella phage vB_KpnS_ZX2]	86%	72.74%	892	QTH79937.1
tail fiber domain-containing protein [Klebsiella pneumoniae]	86%	72.33%	892	WP_216264486.1
hypothetical protein FLA17_gp32 [Enterobacter phage F20]	91%	70.15%	893	YP_009666202.1
tail fiber protein [Klebsiella phage vB_KpnD_Opt-817]	84%	73.22%	892	UGO52585.1
L-shaped tail fiber protein [Klebsiella phage Sugarland]	78%	80.59%	1598	YP_009621065.1
chaperone of endosialidase [Bacteriophage sp.]	91%	60.05%	877	UVM80660.1

L-shaped tail fiber protein [Klebsiella phage Spivey]	51%	80.79%	1103	QBX06900.1
chaperone of endosialidase [Bacteriophage sp.]	88%	60.32%	840	UVM93144.1
L-shaped tail fiber protein [Escherichia phage T5]	77%	76.16%	1396	YP_006961.1
L-shaped tail fibre protein [Escherichia phage T5]	75%	76.16%	1396	AAX12061.1
L-shaped tail fiber protein [Escherichia phage T5]	75%	75.96%	1396	AAU05270.1
long tail fiber [Salmonella phage GEC_vB_N5]	75%	75.35%	1397	QPI15176.1
tail fiber protein [Klebsiella phage Sanco]	53%	83.98%	832	QBZ71179.1
tail fiber [Klebsiella phage Sushi]	53%	83.98%	832	YP_009196676.1
tail fiber protein [Klebsiella phage KpKT21phi1]	54%	80.00%	832	YP_009818762.1
tail fiber protein [Klebsiella phage vB_kpnS-VAC10]	53%	84.17%	780	QZE50957.1
tail fiber protein [Klebsiella phage vB_KpnS-VAC9]	52%	83.86%	770	QZE50307.1
Chain A, L-SHAPED TAIL FIBER PROTEIN [Tequintavirus T5]	44%	80.14%	427	4UW8_A
putative tail fiber protein [Klebsiella phage PWKp17]	84%	54.66%	877	UJD05650.1
putative tail fiber protein [Klebsiella phage BUCT556A]	48%	85.12%	825	UPT53777.1
tail fiber protein [Klebsiella phage UGKSKpnP2]	84%	54.66%	877	CAH1616169.1
tail fiber protein [Klebsiella phage vB_KpnS-VAC8]	54%	71.50%	790	QZE50895.1
TPA: tail fiber domain-containing protein [Klebsiella pneumoniae]	68%	54.62%	674	HBY4082499.1
Chain A, L-SHAPED TAIL FIBER PROTEIN PB8 [Tequintavirus T5]	31%	73.86%	328	5AQ5_A
Chain A, L-SHAPED TAIL FIBER PROTEIN [Tequintavirus T5]	30%	74.24%	294	4UW7_A
tail fiber protein [Pantoea phage vB_PagS_AAS21]	86%	38.05%	946	QCW23758.1
TPA: tail fiber domain-containing protein [Klebsiella pneumoniae]	53%	43.23%	1281	HBR5712471.1
TPA: hypothetical protein [Klebsiella quasipneumoniae]	43%	46.50%	453	HBT5242657.1
tail fiber domain-containing protein [Klebsiella pneumoniae]	55%	42.34%	1374	EIY2407951.1

tail fiber domain-containing protein [Klebsiella pneumoniae]	43%	45.79%	864	EJD6390016.1
tail fiber domain-containing protein [Klebsiella pneumoniae]	43%	46.26%	1195	WP_253885734.1
tail fiber domain-containing protein [Klebsiella pneumoniae]	43%	46.26%	1195	WP_085842367.1
tail fiber domain-containing protein [Klebsiella pneumoniae]	43%	46.26%	1195	WP_088402842.1
TPA: tail fiber domain-containing protein [Klebsiella pneumoniae]	43%	46.03%	1195	HCB0057383.1
TPA: hypothetical protein [Klebsiella quasipneumoniae subsp. similipneumoniae]	53%	42.14%	859	HBW8876201.1
tail fiber domain-containing protein [Klebsiella variicola]	43%	45.79%	1195	WP_101856246.1
hypothetical protein [Staphylococcus epidermidis]	25%	97.35%	191	WP_227954385.1
tail fiber domain-containing protein [Klebsiella variicola]	53%	42.19%	934	WP_264973179.1
tail fiber domain-containing protein [Klebsiella variicola]	53%	42.01%	1279	WP_148675050.1
tail fiber domain-containing protein [Klebsiella variicola]	53%	41.82%	1105	EIV6530553.1
tail fiber domain-containing protein [Escherichia coli]	89%	44.78%	1222	WP_201758357.1
tail fiber domain-containing protein [Klebsiella variicola]	53%	42.14%	1272	WP_110212670.1
tail fiber domain-containing protein [Escherichia coli]	90%	43.94%	1221	EFA4692338.1
phage tail fiber protein [Sodalis glossinidius]	44%	41.90%	701	WP_041866876.1
tail fiber domain-containing protein [Pantoea agglomerans]	44%	44.83%	877	WP_115765313.1
hypothetical protein phiW14_00062 [Klebsiella phage phiW14]	41%	73.71%	726	UEW68502.1
TPA: tail protein [Siphoviridae sp.]	44%	41.88%	579	DAV55934.1
hypothetical protein [Klebsiella phage vB_KvaS_F2M1D]	21%	81.99%	897	UNA05281.1
hypothetical protein [Escherichia coli]	35%	44.86%	334	NBY92354.1
non-contractile tail fiber protein [Klebsiella phage vB_KpnS-VAC70]	16%	96.23%	815	UEW68236.1
hypothetical protein [Staphylococcus epidermidis]	17%	89.94%	298	MCC3754327.1

chaperone of endosialidase [Bacteriophage sp.]	40%	46.19%	1221	UVY41953.1
tail fiber protein [Klebsiella phage IME268]	29%	72.00%	818	QYC96849.1
tail fiber protein [Klebsiella phage JY917]	17%	90.12%	812	YP_009903186.1
tail fiber protein [Klebsiella phage VLCpiD7b]	17%	88.51%	793	UVX31064.1
tail fiber protein [Klebsiella phage Sin4]	21%	75.36%	785	YP_009903484.1
tail fiber protein [Klebsiella phage Shelby]	16%	92.73%	446	YP_009903407.1
chaperone of endosialidase [Bacteriophage sp.]	46%	45.16%	1323	UWG30148.1
hypothetical protein BMKJMICM_00042 [Klebsiella phage vB_1086]	17%	89.35%	845	UJQ43178.1
tailspike protein [Stenotrophomonas phage vB_SmeS_BUCT705]	17%	89.35%	845	UNY50363.1
